# What have we learned about COVID-19 volunteering in the UK? A rapid review of the literature

**DOI:** 10.1186/s12889-021-11390-8

**Published:** 2021-07-28

**Authors:** Guanlan Mao, Maria Fernandes-Jesus, Evangelos Ntontis, John Drury

**Affiliations:** 1 University of Sussex, Brighton, UK; 2 Canterbury Christ Church University, Kent, UK

**Keywords:** COVID-19, Volunteering, Rapid review, Self-isolation, Community champion, Community engagement, Mutual aid

## Abstract

**Background:**

Community engagement and volunteering are essential for the public response to COVID-19. Since March 2020 a large number of people in the UK have been regularly doing unpaid activities to benefit others besides their close relatives. Although most mutual aid groups emerged from local neighbourhoods and communities, official public institutions also fostered community volunteering, namely through the community champions scheme. By considering a broad definition of COVID-19 volunteering, this article describes a systematic review of the literature focused on one broad question: What have we learned about COVID-19 volunteering both at the UK national level and the more local community level?

**Methods:**

A rapid review of the literature in peer-reviewed databases and grey literature was applied in our search, following the PRISMA principles. The search was conducted from 10 to 16 of October 2020, and sources were included on the basis of having been published between January and October 2020, focusing on COVID-19 and addressing community groups, volunteering groups, volunteers, or community champions in the UK.

**Results:**

After initial screening, a total of 40 relevant sources were identified. From these, 27 were considered eligible. Findings suggest that food shopping and emotional support were the most common activities, but there were diverse models of organisation and coordination in COVID-19 volunteering. Additionally, community support groups seem to be adjusting their activities and scope of action to current needs and challenges. Volunteers were mostly women, middle-class, highly educated, and working-age people. Social networks and connections, local knowledge, and social trust were key dimensions associated with community organising and volunteering. Furthermore, despite the efforts of a few official public institutions and councils, there has been limited community engagement and collaboration with volunteering groups and other community-based organisations.

**Conclusions:**

We identified important factors for fostering community engagement and COVID-19 volunteering as well as gaps in the current literature. We suggest that future research should be directed towards deepening knowledge on sustaining community engagement, collaboration and community participation over time, during and beyond this pandemic.

## Background

The COVID-19 pandemic has provoked a remarkable surge in volunteering and community support around the world [1, 2]. Prominent manifestations of this outpouring of community spirit within the UK include the rise of so-called mutual aid groups, volunteer-led initiatives where individuals from a particular area group together to meet community needs [3]. Over 4000 local groups have formed over the course of the pandemic, with as many as three million participants [3, 4]. On a national level, the National Health Service (NHS) volunteer responders’ scheme was able to recruit over 750,000 people in 4 days, three times the initial target [5]. Additionally, some local authorities further promoted community champions programmes during the pandemic [6].

Community champions are trained and supported volunteers whose purpose is to help improve the health and wellbeing of their communities. Community champions are closely connected with their communities and can include local leaders and individuals within community organisations [7]. They share information, motivate and empower people to get involved in health-promoting activities, create groups to meet local needs, and direct people to relevant support and services [8]. Community champions may also engage in more active involvement with authorities, such as consultation to provide insight into community needs and involvement in the planning, design, implementation and evaluation of services [9].

Evidence from past pandemics suggests that such community involvement is crucial in fostering public health in pandemic conditions and reducing contagion, with community action groups playing a key role in the success of campaigns against Ebola and AIDS [10–12]. In the case of the COVID-19 pandemic, in many countries (though not all – see [13]), one of the key Non-Pharmaceutical Interventions (NPIs) that has largely been left to communities themselves to manage is self-isolation [14]. This is a behaviour that requires support from others to be achieved – unlike hand hygiene and mask-wearing, for instance – and therefore exemplifies the essential role of volunteers in local communities [15, 16].

While adherence rates in the UK for most NPIs have been high [17], surveys have consistently estimated that the number of people self-isolating for the full 10–14 days required^1^ is less than 50% and sometimes as low as 18% [18–21]. One of the main reasons found for breaking self-isolation is going to the shops for provisions [22]. But while community volunteers have been critical in supporting self-isolation, it is important to understand the other contributions they have made to the pandemic response, who is involved, and the factors that support or impede their activities.

In this study, we focus on a broad definition of COVID-19 volunteering, to capture the multiple ways people engaged in community support and mutual aid groups, as well as in community champions programmes. The definition of volunteering we adopt encompasses both informal volunteering, defined by National Council for Voluntary Organisations (NCVO) as “giving unpaid help as an individual to people who are not a relative”, and formal volunteering, or “giving unpaid help through a group, club or organisation” [23].

We report a rapid review of the literature that addresses the following broad question: What have we learned from COVID-19 volunteering both at the UK national level and the more local community level? Answering this question may also illuminate political, organisational and psychological aspects of COVID-19 volunteering that will be useful for responses to future disease outbreaks.

## Methods

A rapid review of literature was applied in our search. Rapid reviews are a useful form of producing information in a timely manner [24]. A multi-faceted approach, following the Preferred Reporting Items for Systematic Reviews and Meta-Analyses (PRISMA) principles [25], was adopted in order to scope the rapidly evolving literature base for the topic. This review was not registered.

Searches were conducted in peer-reviewed databases and grey literature. The inclusion of grey literature in public health reviews can help advance the understanding of what and how interventions are being implemented [26]. As grey literature is not controlled by commercial publishing organisations, the information is published when a particular phenomenon is occurring. This is particularly useful for applied researchers and practitioners, in disasters and pandemic conditions such as COVID-19, where relevant and timely information is urgently needed.

### Search strategy

The search was applied to six databases: ScienceDirect, University of Sussex Library, APA Psycnet, Wiley Online Library, PubMed and SocArXiv. These databases were selected based on their coverage of the topic. For grey literature, a search was first conducted via Google Advanced to identify a number of think tanks, governmental, and third sector organisations which had been conducting research relevant to COVID-19 volunteering. We then searched the websites of these organisations for all relevant sources using a Google site search. This same process was repeated for academic websites, blogs and research networks. Finally, we conducted another web search using Google Advanced for sources not connected to such wider organisations but which were nonetheless from reliable authorities and met our inclusion criteria.

The search was conducted between October 10 to 16, 2020, using the following keywords: (“COVID-19” OR “Coronavirus”), AND (“volunteering” OR “mutual aid” OR “community”, “community engagement”, OR “community champions”). Records were included only if they were published between January and October 2020, focused on COVID-19 and addressed community groups, volunteering groups, volunteers, or community champions in the UK. We considered many source types, including published peer reviewed articles, reports, briefings, blogposts, newspaper articles, and online media relevant to research questions. Only English sources were considered.

### Analytic procedure

A narrative synthesis approach [27] was adopted due to the heterogeneity of the eligible sources. Data extracted from each source included the title, authors, publishing organisation, setting, sample size (if relevant), and data collection period, as well the content or text itself. The content of each source was analysed using techniques based on thematic analysis [28], whereby the researcher approaches the text with certain research questions in mind and aims to organize data into patterns of meaning or interpretative themes in relation to these questions. This organization is an iterative process, as the initial categorization of a piece of data may be adjusted as themes are merged (if themes are too similar) or split (if the material included is too diverse). The present analysis involved one of the authors (GM) reading through each source, highlighting, making notes, and compiling findings, case studies and statistics relevant to the research question. During this process, material from within the sources was organised and placed in relation to other pieces under a preliminary set of meaningfully distinguishable themes, distinguishing such issues as profile of volunteers, types of activities, and models or organizing. A preliminary analysis was written. The other authors (MFJ, JD and EN) read through this preliminary analysis and discussed the relevance of each theme in relation to the research question, identifying possible areas of overlap and redundancies, before reaching a finalised set of five superordinate themes, three of which were further subdivided into two sub-themes each.

### Risk of bias

Risk of bias was measured by one of the authors (GM) using the Mixed Methods Appraisal Tool (MMAT) [29]. Studies were evaluated on five dimensions, which differed depending on the study method. Studies were rated as good quality if they scored four or more out of five; moderate quality if they scored three out of five; and poor quality if they scored two or less out of five.

## Results

**Fig. 1:**
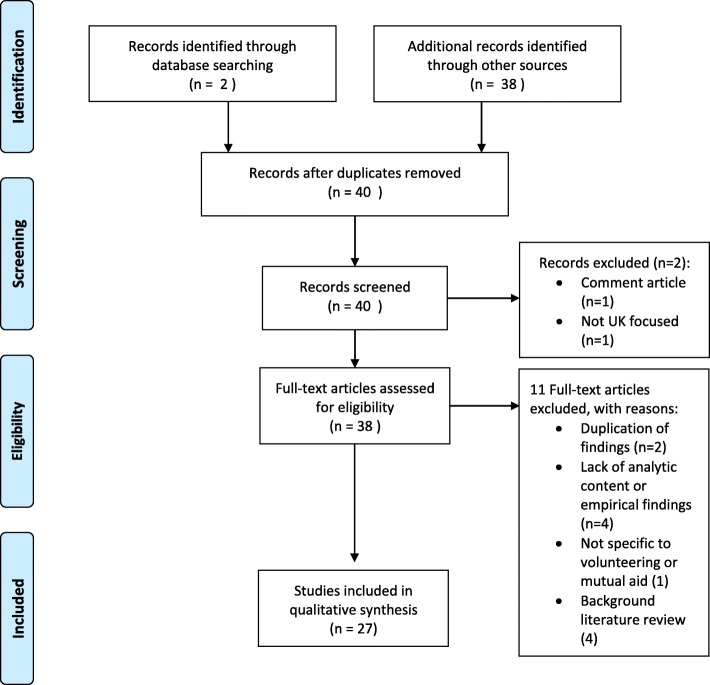
Flow Diagram representing the selection process of articles

After initial screening, a total of 40 relevant sources were identified, including two from published literature databases, 29 from governmental and third sector organisation websites, think-tanks, six from Google Advanced, and three from academic websites, blogs and research networks. After all of these sources were assessed for eligibility, 27 were included in the final qualitative synthesis (Fig. 1).

Table 1 reports the characteristics of the sources analysed, including the type of source, the setting, the sample size (if applicable), the study design, the process followed in data collection, and a short summary of the major findings. Most eligible sources identified can be considered grey literature and were produced by civil society organisations. From the 27 sources included in the qualitative analysis there were: 13 reports, three briefings, five blogposts, two newspaper articles, two websites entries, and two peer reviewed journal articles. Fifteen sources used primary data, 10 secondary and two sources were based on both types of data. A few sources were based on large surveys with volunteers, but the majority focused on qualitative forms of enquiry (e.g., interviews, conversations) with volunteers, stakeholders, or organisations. Most of the sources focused on the national context or on a large set of regions.

**Table 1:** Characteristics of the included sources

Authors and Year	Item type	Organisation	Type of source	Setting	Sample size (if relevant)	Study Design	Data collection period	Findings
Alakeson, V, Brett, W (2020) [30]	Report	Power to Change	Combination of primary + secondary	UK	Not stated	Collective input from stakeholders	Not stated	• Mutual aid works best at the micro level. • Mutual aid at scale requires community organisations • Community organisations have changed quickly to meet local need. • Bigger institutions rely on community organisations to respond well.
Britain Thinks (2020) [31]	Report	West Midlands Recovery Coordination Group	Primary	West Midlands	36	Series of discussions with Citizen’s panel of local residents	03/06/2020–02/07/2020	• Priorities include getting back to normal safely, healthcare, mental health, education, employment, promoting and supporting business.
Felici (2020) [32]	Blog post	Bennett Institute for Public Policy	Secondary	UK	N/A	Statistical analysis of geographic density of mutual aid groups	27/03/2020	• There is a positive correlation between density of mutual aid groups and measures of socio-economic advantage.
Gardner, 2020 [33]	Newspaper article	The Telegraph	Secondary	UK	N/A	N/A	07/04/2020–16/04/2020	• NHS Volunteer army given fewer than 20,000 tasks since launch.
Jones et al. (2020) [34]	Peer-reviewed article	University of the West of England	Primary	Bristol	539	Survey	06/04/2020–20/04/2020	• Members of Covid-19 support groups provided a wide range of support and cited a variety of successes and failures. • 46.7% of respondents wanted to become more involved in the neighbourhood in the future. • With respect to most measures there were no differences in the characteristics of support between respondents in areas of high and low deprivation.
Kavada (2020) [35]	Blog post	Open Democracy	Secondary	UK	N/A	N/A	N/A	• The creation of “micro-groups” in specific areas helped to create trust • Mutual aid groups used a variety of digital tools to organise. • The decentralised organising model of mutual aid groups is faster and more agile than the centralised model. • Mutual aid groups may become involved in political campaigns regarding the broader impact of the pandemic.
Local Government Association (2020) [36]	Website	Local Government Association	Secondary	N/A	N/A	N/A	N/A	• Large repository of case studies of good council practice in response to Covid-19.
Locality, 2020 [37]	Report	Locality	Primary	Berwick, Grimsby, Norfolk, Holburn, Levenshulme, Hackney, Coventry	7 case study interviews; 57 survey responses	Case study interviews with community leaders; qualitative survey; member roundtables; contributions from local authority leaders	Not stated	• Existing social infrastructure was crucial to the crisis response. • The crisis has created new and improved partnership working between community organisations and the public sector. • Community organisations have connected different layers of response. • Managing new volunteering capacity came with challenges • Community organisations have adapted at pace but require support for the future.
Mak & Fancourt, 2020 [38]	Peer-reviewed article	UCL	Primary	UK	31,890	Survey of Covid-19 volunteers	21/04/2020–03/05/2020	• Three types of Covid-19 volunteering identified: formal volunteering, social action volunteering, neighbourhood support. • Volunteering was associated being female, living with children, living rurally, having higher educational qualifications, and higher household income. • New groups identified as likely to volunteer were people with a physical or mental health condition. • The predictors of volunteering during the pandemic may be slightly different from other non-emergency period.
McCabe, A., Wilson, M., & MacMillan, A. E. (2020) [39]	Briefing	Local Trust	Primary	26 areas in England	Not stated	“Learning conversations” with residents, community activists and workers; Interviews with Big Local reps	04/2020–06/2020	• Communities have been resourceful in developing creative ways of bringing resources together to respond quickly to community need, using technical knowledge to implement alternative ways of working; applying local knowledge to meet immediate needs; promoting acknowledged roles.
McCabe, A., Wilson, M., & Macmillan, R. (2020) [40]	Report	Local Trust	Primary	26 areas in England	317 conversations; 20 Interviews	“Learning conversations” with residents, community activists and workers; Interviews with Big Local reps	04/2020–09/2020	• Community responses to the immediate crisis have varied significantly. • Most communities have moved on from an initial crisis response and are looking ahead. • An established community-led infrastructure underpins an effective community response.
McCabe, A., Wilson, M., & Paine, A. E. (2020) [41]	Briefing	Local Trust	Primary	26 areas in England	Not stated	“Learning conversations” with residents, community activists and workers; Interviews with Big Local reps	04/2020–10/2020	• A new cohort of volunteers has emerged who are often younger and on the furlough scheme. • Engagement at grassroots level has been more effective than command-and-control. • Factors identified as important in the successful retention of volunteers include clear boundaries, permissions, social rewards, nurturing relationships, feeling valued.
NewLocal, 2020 [42]	Report	New Local	Primary	UK	94	Survey of local government leaders, chief executives and council mayors	9/04/2020–21/04/2020	• 95.6% of respondents highly value the contribution of community groups in their council’s effort to tackle Covid-19 (47.4% very significant, 48.2% significant). • Council chiefs are more confident there is community cohesion in their area, with confidence levels at 71.9%
NHS England (2020) [5]	Website	NHS	Primary	N/A	N/A	N/A	27/03/2020–29/03/2020	• The NHS Volunteer responders initiative has recruited 750,000 people in 2 days.
O’Dwyer (2020) [43]	Blog post	Kingston University	Primary	UK	854	Survey of mutual aid group members	Not stated	• Participants are predominantly white, female, middle class, and more political than average. • Participants were generally left wing but tended not to see their mutual aid groups as political.
Scottish Government (2020) [44]	Report	Scottish Government	Primary	Scotland	62	Qualitative survey of community organisations	15/05/2020–27/05/2020	• The pandemic has prompted large changes to the operations of respondents. • Covid-19 has presented increased demands, most prominently the provision of food. • Half of participants mentioned improved partnership working. • Priorities for the future include mental health support, employment, building a wellbeing and low carbon economy, tackling inequalities, capitalising on rise in community support.
Spratt (2020) [45]	Newspaper article	The i	Secondary	N/A	N/A	N/A	N/A	• ACORN have seen a large increase in membership over Covid-19. • ACORN have been holding “eviction resistance” bootcamps to tackle the rise in evictions.
Taylor and Wilson (2020) [46]	Report	Community Organisers	Combination of primary + secondary	UK	Not stated	Literature review; Interviews with people involved in community organising	Not stated	• Communities with an organising history were able to respond quickly and flexibly as previous community organising activity meant that local people were already connected. • Vast majority of support provided was “practical help” including delivering food, collecting prescriptions, making check-in calls. • Organisers adapted to the need to go online through use of technology but also developed methods for reaching the digitally excluded. • Community organisers have supported residents to challenge government policies and practices.
Tiratelli (2020b) [47]	Blog post	New Local	Secondary	N/A	N/A	N/A	N/A	• The activity of mutual aid groups declined sharply when lockdown eased. • Many mutual aid groups are dormant but the infrastructure they have created remains. • Mutual aid groups may spring back into action if a second lockdown occurs.
Tiratelli & Kaye, 2020 [48]	Report	New Local	Combination of primary + secondary	UK	Not stated	Literature review; Observation of mutual aid groups’ social media; Interviews with mutual aid participants	Not stated	• Some mutual aid groups form spontaneously and others as outgrowths from existing community projects • Digital infrastructure was important • The furlough scheme led to a different demographic profile of volunteers than usual • Activities of mutual aid groups have evolved to encompass wider social support over time • Councils should adopt facilitative approaches to working with Mutual Aid groups rather than controlling or indifferent approaches.
Tiratelli, 2020a [49]	Report	New Local	Combination of primary + secondary	UK	Number of interviews not stated	Literature review; Interviews with experts on the topic of community mobilisation	Not stated	• Community engagement is a shallower process than community mobilisation. • Approaches to community mobilisation can focus on different units: individuals, groups, places, and services. • Public bodies interested in community mobilisation need to: take a facilitative approach; listen to communities; build something that was not there before; have clear goals.
Volunteer Scotland, 2020 [50]	Report	Volunteer Scotland	Primary	Scotland	4827	Survey of charities	05/05/2020–15/05/2020	• 37% of charity volunteers have been unable to work during COVID-19.
VSF (2020) [51]	Report	Primary	Secondary	N/A	13	Collective input from Volunteering Support Fund projects	Not stated	• Many projects shifted their operations to the online world. • Support was offered to volunteers and service users with using technology. • Many projects reported increase in volunteer recruitment. • Projects adapted to respond to the pandemic, some changing their focus entirely.
Wein (2020) [52]	Report	Dignity Project	Primary	UK	182	Survey of mutual aid group members	11/05/2020–30/05/2020	• In 53% of groups a small group of people made the decisions whilst 33% had more consensual decision-making. • Support on technology and communication was most desired by groups (32%) • 83% of respondents intended to take some political action in the coming year and 49% will take at least 3 actions. • Demographics: 65% female, median age 48, 48% earned less than median income, better educated were overrepresented.
Wilson, McCabe & MacMillan (2020) [53]	Briefing	Local Trust	Primary	26 areas in England	Not stated	“Learning conversations” with residents, community activists and workers; Interviews with Big Local reps	04/2020–08/2020	• Informality has assisted the speed and flexibility of responses to Covid-19 but scaling is an issue. • Organisations have been mixing both formal and informal ways of working. • Pre-existing community infrastructure has facilitated the co-ordination of responses to Covid-19.

### Risk of Bias analysis

Using the MMAT, the mean average risk of bias score was 2.8 from a maximum of 5, excluding those which did not meet the initial screening criteria (where a higher score means lower risk of bias). Based on available information (see risk bias assessment at https://osf.io/6vbpu/?view_only=025e3891c2d845e0af9271bed5c06e53), 5 studies were rated as high quality, 6 as medium, and 7 as low. Many studies did not describe their methodology in sufficient detail for specific evaluations to be made: in these cases, a rating of “Can’t Tell” was given. The risk of bias score for each study was determined according to the number of methodological quality criteria met, with each “Yes” counting as 1 and each “No” or “Can’t Tell” counting as 0. Twenty-one studies did not meet the initial screening criteria: these consisted of reports, news articles, and collections of case studies which did not have a clear research question. These studies were nonetheless included as they provided some evidence of what volunteers had been doing during COVID-19 volunteering and how, and therefore aided our rapid review in addressing the research question.

### Overview of findings

In the following sections we will describe the findings of this review by focusing on the five major topics: volunteering activities (subdivided into “needs addressed by volunteers” and “adapting through usage of digital tools”); models of volunteering; volunteer profiles (subdivided into “shifting demographics” and “predictors of volunteering”); successes, challenges, and determinants of effectiveness; interactions with authorities (subdivided into “collaboration with local communities” and “consultation with local communities”).

### Volunteering activities

#### Needs addressed by volunteers

Studies reviewed indicate that delivery of essentials such as food and prescriptions dominated early efforts in COVID-19 volunteering [39]. A second type of activity which became increasingly common as the first ‘lockdown’ wore on was combating social isolation through activities such as provision of arts and crafts packs, telephone support, and online social activities [39]. After the first ‘lockdown’ (23rd March – June-July 2020), there was an increasing shift towards volunteering activities that address the wider impact of the pandemic on other areas such as employment, social benefits, mental health, domestic abuse, and homelessness [35, 39]. There is also some evidence that COVID-19 volunteers became involved in wider political campaigns [35, 52]. Wein [52] found that 83% of mutual aid participants intended to take part in some form of political action in the coming year, with 64% likely to sign petitions and 47% expecting to contact a politician. Information about the Black Lives Matter movement has reportedly been circulating in WhatsApp groups, whilst activists associated with mutual aid groups staged an action outside the house of Dominic Cummings [35]. In one case study described by McCabe et al. [41], volunteers who witnessed the living conditions of those they were helping developed a collective action approach to addressing poor housing conditions. On an organisational level, ACORN, a community union which organised mutual aid networks around the country, has worked to divert many of its volunteers from community support to eviction resistance campaigns [45]. Importantly, the activities of these volunteers expose the insufficiency of public services [48], as they are serving to meet needs which are otherwise unmet by public services.

#### Adapting through usage of digital tools

There is also evidence that COVID-19 volunteers adapted not just to changing needs but to external circumstances which made traditional forms of organisation difficult. This was most notable in the shift from offline to online volunteering. In many cases, existing voluntary organisations and projects adapted their services by transferring to digital infrastructure, often at a rapid pace [37]. Some groups entirely recreated their activities online, such as a weekly Facebook-based interactive youth club, or a lunch club for people living with dementia held via Zoom [40, 51]; others utilised a plethora of online tools and technologies to complement offline activities [46]. Whilst WhatsApp was one of the most popular organising platforms, some groups adopted more streamlined services such as Slack. Platforms such as Zoom and Skype were used for group calls; Google Docs for meeting minutes; and Google Sheets for compiling databases of volunteers and requests [35].

Whilst such methods may raise a problem of digital exclusion, many groups explicitly sought to tackle this possibility by combining online volunteering with offline methods such as mass leafleting [34]. Other projects (for example Skills Enterprise in East Ham) offered digital training sessions, as well as providing tablets and phones to those on their programmes [37, 51]. The crisis has demonstrated the adaptability and resourcefulness of volunteers and community organisations, who have effectively adjusted to changing conditions as the UK continues to pass through different phases of the pandemic.

### Models of volunteering

The onset of lockdown saw an outpouring of community spirit and voluntarism, channelled in a huge variety of ways [39]. Whilst in some areas volunteering activity surfaced spontaneously, in other areas this activity emerged as an outgrowth of existing networks, community projects, and organisations [48]. In many cases such organisations shifted their activities rapidly to COVID-19, mobilising volunteers and relationships with other local groups to create local support schemes [37]. For example, Homebaked in Anfield, a community bakery, closed down much of its traditional operations and started baking 50 to 70 loaves a day, which it provided to the local food bank and community centre [30]. Focusing precisely on the models and predictors of volunteering, a large survey with 31,890 adults in the UK identified three types of volunteering during COVID-19 [38]. The first, ‘formal volunteering’, included volunteering in formal and pre-existing structures and organisations. The second type, ‘social action volunteering’ was described as more oriented to broad fundraising and donation campaigns. Finally, ‘neighbourhood support’ involved providing support locally (e.g., shopping or cooking meals for others) [38].

Other authors have noted the emergence of two models of volunteering coordination during the pandemic [35, 54]. On the one hand, a decentralised model, where information and decision-making are dispersed among members. On the other hand, a centralised method of command-and-control. These authors have generally argued for the superiority of the former model in terms of its speed, democratic nature, and ability to meet the needs of those excluded from other services [35, 54]. Kavada [35] compared the model of mutual aid groups to the NHS volunteer responders service. The formal nature of the NHS scheme meant that the identities of all volunteers had to be carefully checked, leading to delays in assignment. Furthermore, the service only served UK inhabitants who registered as vulnerable, excluding those unwilling or unable to register formally [35]. In contrast, mutual aid groups did not engage in verification of volunteers, and supported anyone who was self-isolating, allowing them to meet the needs of their communities more effectively [35].

However, one common challenge reported by many mutual aid groups was a lack of leadership, where people were keen to offer services but were not willing to take the initiative [48]. Equally, those engaged in more informal ways forms of supporting their neighbours also frequently reported the same challenge regarding reaching vulnerable groups, with help either lacking focus or being limited to those cases already known [34, 39]. Furthermore, categorising volunteer activity as hierarchical or non-hierarchical, centralised or decentralised, formal or informal seems an oversimplification. In reality, most organisations combined elements of both approaches [53]. Many groups preserve a ‘private layer’ of interaction for ‘core’ members and organisers [48], and group administrators were able to participate in a closed Facebook group to exchange tactics [35]. Despite this caveat, sources reviewed suggest that the pandemic has prompted a qualitative shift in volunteering around the country, with traditional formal organisations such as charities losing a large bulk of their volunteers whilst informal associational models are thriving [50].

### Volunteer profiles

#### Shifting demographics

The conditions of the pandemic should arguably pose a challenge for volunteering efforts given its high risk to the elderly, normally the demographic most likely to volunteer regularly [23]. However, the present circumstances appear to have led to the emergence of a new volunteer workforce. Results of surveys [43, 52] suggest that the average age of COVID-19 mutual aid group members was 48. Another study [38] reported a slightly higher mean age (52 years old), concluding that older people were more likely to participate in volunteering than younger people, particularly in activities that involve providing local neighbourhood support. More generally, mutual aid groups appear to be concentrated in areas with large numbers of working-age people, a clear consequence of the government’s furlough scheme [48].

#### Predictors of participation

Volunteers were composed of more women than men [43, 52], especially in neighbourhood volunteering and social action volunteering [38]. Whilst this is in line with general trends [23], it may also represent an extra caring responsibility at a time when women were already shouldering the burden of increased domestic labour.

There were also early indications that wealth and class played a role in participation. An analysis by Felici [32] of voluntary support networks across the UK revealed a positive correlation between the density of voluntary groups in an area, which is one of the manifestations of social capital, and measures of socioeconomic advantage, as well as well-being. In turn, Wein [52] argued that participants themselves are not necessarily wealthy. His survey indicates that 48% of volunteer households had an income of less than £30,000 and 30% above, compared to the national median of £29,600. Similarly, Mak and Fancourt [38] found that income predicted engagement in social action volunteering but did not predict other types of volunteering. However, it is important to remember that the resources and tactics available to these participants, and therefore the overall effectiveness of their participation, may not be the same. Indeed, a report by Taylor and Wilson [46] based on the experiences of community organisers found that whilst most affluent communities organise themselves, communities within more deprived areas often need more support but lack access to resources. In this regard, one participant in a mutual aid group from a poor rural area suggested that tactics such as crowdfunding would not be effective in rural areas [48]. Nevertheless, Mak and Fancourt [38] found that whereas people who lived in rural areas were more likely to engage in formal and neighbourhood volunteering, there were no differences in terms of social action volunteering.

In terms of psychosocial and personality predictors, the only study that addressed these predictors found that personality traits (e.g., agreeableness, extraversion), but also social support and social networks were associated with engagement in all types of voluntary work during the pandemic [38]. Interestingly, Abrams et al. [55] found that compared to people who had not volunteered, those who were volunteers during COVID-19 reported higher trust in people to follow guidelines, trust in government, and compassion for people in the local area. They also scored higher on connection to family, friends, colleagues and neighbours, and connection to their local area [55].

### Successes, challenges, and determinants of effectiveness

By delivering vital services to vulnerable individuals in the early days of lockdown whilst traditional public services struggled to respond effectively, mutual aid groups undoubtedly played a life-saving role in the UK’s COVID-19 response [48]. Such groups have also generated new partnerships, networks and knowledge, which may serve as a long-term resource in the second wave [49]. In terms of community and voluntary organisations generally, 95% of council leaders and chief executives saw community groups as being significant or very significant in their COVID-19 response [42].

However, volunteer groups have also faced many challenges. Many have found it hard to sustain the morale and enthusiasm of volunteers over time, with the activity of many groups declining sharply once lockdown started to ease [49]. Other volunteering schemes found it hard to generate sufficient demand or faced high bureaucratic procedures that delayed their interventions [39]. For example, the length of time it took for volunteers to hear back from the NHS Volunteer Responders Scheme caused initial enthusiasm to dissipate [37]. Later data revealed that in the first week of the scheme, the 750,000 volunteers were given fewer than 20,000 tasks between them [33]. By contrast, smaller mutual aid groups who attempted to scale up their operations beyond street level often found that they were lacking in organisation, coordination, local relationships, and trust [30]. This was the case with a group formed in Dalston, London, which quickly attracted hundreds of volunteers but was unable to attract requests for support due to distrust from the local community [30].

In terms of sustaining volunteering, factors identified by groups as being important to successful retention of volunteers included: not asking volunteers to engage in activities they are uncomfortable with; allowing volunteers to say no; providing social rewards; nurturing relationships with volunteers; and recognising the contributions of volunteers [41]. Moreover, a common theme emerging from the research reviewed is that effective and rich responses are underpinned by ‘community-led infrastructure’, understood as community leadership, trust, relationships with agencies, and access to funds [39]. In particular, many community organisations have been able to play a coordinating role by providing smaller mutual aid groups with the infrastructure, systems, and resources required, as well as acting as a communication bridge between groups and local authorities [37]. Local knowledge has also been important in responding to the needs of groups not covered by government schemes, such as homeless people or families with young children [53]. For example, the Hastings Emergency Action Response Team (HEART) has been able to coordinate over 900 volunteers, using their local knowledge to identify needs [30]. Additionally, the nationwide union ACORN was able to set up support systems in nine cities by mid-March. After years of organising and campaigning, ACORN already had an engaged existing membership in each city and well-developed organisational structures [46].

### Relationships with authorities

#### Authorities collaborating with local communities

The above discussion naturally raises the question of how authorities can best support local community-led infrastructures. In some cases, this was achieved through the COVID-19 Community Champion scheme [36, 56]. These volunteers were given the latest information about COVID-19 and were asked to share this information in their community, whilst feeding back which communications are effective, and which are not [56].

Councils which have successfully implemented the scheme include Newham Council, which have recruited more than 500 people to date [36]. Champions receive messages through WhatsApp or email most days, including infographics which are available in a variety of languages. Among other things, they are given a badge and are included in a WhatsApp group so that they can share advice and support one another. Newham Council have now supported more than 30 other councils to develop their own programmes [36]. Despite such successful case studies, as of the time of writing no systematic report or review has been published regarding the impact of this scheme. It is worth noting, however, that the role of the community champions is not far from what many mutual aid volunteers took it upon themselves to operate in the early days of the pandemic. As reported by Jones and colleagues [34], 57% of volunteers in mutual aid groups also supported their neighbours by providing information about the virus.

Moreover, Tiratelli and Kaye [48] distinguish between three types of local council approaches to community organisations and mutual aid groups: micromanage, indifferent, and facilitative. In the micromanaged approach, councils seek to control the efforts of volunteers and community organisations, issuing orders in a prescriptive language of ‘should’ and ‘must’, an approach which has caused participants to view local government as an obstruction [48]. In the indifferent approach, councils fail to support such groups and refuse to collaborate with them, an approach which potentially hobbles volunteering and damages public trust. For example, Locality [37] members have reported a lack of information sharing and joint planning, an approach which has led to duplication and confusion, as well as a lack of support in accessing funding. These two approaches are contrasted with the facilitative approach, in which local authorities find ways to support communities without smothering them (e.g., by providing practical help such as supplying mobile phones and card readers; proactively connecting volunteers with existing networks and other groups; or providing spaces and infrastructure to help groups organise) [48]. In Bristol, the community hub Wellspring Settlement was able to develop a system with the local authority to have volunteers Disclosure and Barring Service (DBS) checked in 24 h [30]. As councils that have made concerted efforts in community engagement are the ones that have best facilitated their local mutual aid groups [48], councils should seek to give community organisations the freedom to operate whilst providing practical support and advice when needed.

#### Authorities consulting local communities

Some authorities have sought not only to collaborate with local communities involved with COVID-19 volunteering but to consult them regarding their priorities and needs going forwards. To our knowledge, the COVID-19 period has seen only two completed consultations of local communities thus far. These consisted of one by The West Midlands Combined Authority to guide its COVID-19 recovery [31], and one by the Scottish Government on the impact of COVID-19 on community organisations and their priorities for recovery [44]. The panel for the West Midlands Combined Authority agreed on six priorities for the recovery: getting safely back to normality; ensuring clear guidance is provided as communities move out of ‘lockdown’; a strong healthcare system, including mental health; preparing children to go back to school in a supportive environment; creating new jobs and training with an emphasis on apprenticeships and entry-level jobs; promoting and supporting businesses, especially smaller and local businesses. Some of the priorities identified in the Scottish consultation involve supporting mental health; limiting the impact of future cuts and reduced services on communities; addressing employment issues; a low carbon recovery; tackling inequalities, and capitalising on the rise in community spirit [44]. As of November 2020 Bristol City Council are also conducting a multi-stage engagement process which will involve a survey, online forum, and citizen’s assembly regarding the city’s COVID-19 recovery, all of which will feed into its overall recovery plan [57].

Whilst consultation practices are important, it is also worth acknowledging their limitations. Tiratelli [47] argues that such forms of engagement are often merely a pro-forma procedure with non-intention of handing over power to the communities in any meaningful way. Such approaches need to be combined with a meaningful project of community mobilisation, which builds strong coalitions, leadership, and engenders local communities with the belief that they can enact real change [47]. It is precisely this mass mobilisation which has proved so invaluable in a time of crisis, and if properly tended to may lead to even greater things.

## Discussion

This review focused on the nature and dynamics of COVID-19 volunteering as well as on how community engagement was fostered during the COVID-19 pandemic in the UK. First of all, it is worth noting that this rapid review has several limitations namely related to the period covered. While this study helps to understand patterns of COVID-19 volunteering in the UK during the early phases of the pandemic, it does not allow us to respond to our research question completely. New research has been conducted and published since we developed this review (e.g. [58]), and the COVID-19 pandemic keeps challenging communities and community groups. Nevertheless, important conclusions can be drawn from our study, specifically related to the emergence and development of the COVID-19 volunteering during the first year of the pandemic.

In terms of what we have learned from COVID-19 volunteering, we conclude that COVID-19 models of volunteering were diverse, not only in terms of modes of organising (e.g., more or less horizontal, formal or informal) but also in terms of the activities that were developed [35, 39]. Since the COVID-19 outbreak, communities across the country were able to mobilise and organise into multiple and diverse forms of community action and support. Some existing groups changed their focus and started to support their local neighbourhoods. People without previous experience of volunteering set up informal support groups from scratch in their local areas. Thousands of mutual aid groups were created, and many people were active helpers in providing information about COVID-19, shopping, packing and delivering food, fundraising and making donations, collecting prescriptions, dog walking, and offering emotional support through telephone helplines, among others [4, 37, 39].

In terms of the profile of the volunteers, the studies reviewed suggest that the demographic makeup of COVID-19 volunteers partly reflected pre-existing trends and inequalities, by showing that women, working-age people, and middle-class people were more engaged in volunteering than other demographics [38, 43, 48, 52]. In addition, higher levels of social support, cohesion and trust, and pre-existing social networks were important dimensions in explaining the emergence of COVID-19 volunteering and also the profile of volunteers [32, 38, 55]. However, is still unclear whether these dimensions reflect the different profile of the volunteers or some consequences of engaging in volunteering during a crisis. Further research is needed to better understand the profile of volunteers during the outreach, as well as whether the demographic and geographic distribution of volunteering may simply reproduce and even reinforce the existing inequalities exacerbated by COVID-19. If so, any governmental response should address the underlying socioeconomic disadvantages which hinder effective voluntary action [32].

Importantly, this review indicates that volunteering practices changed since the first UK ‘lockdown’ (23rd March – June-July 2020), and that support groups adjusted their activities and actions over time [37]. Overall, our review suggests that communities and groups began to reorient themselves beyond the temporally bound demands of the pandemic context, and towards more fundamental structural demands. We argue that this shows not only that groups are willing to continue to provide community support but that they are also able to change their focus and adapt to new needs and challenges. The ability to adjust and adapt the activities of the group is very promising, and future studies should look at COVID-19 volunteering patterns over time.

Furthermore, our review suggests that groups strategically developed activities to promote volunteer well-being and avoid overloading them with tasks [41]. Past research suggests that such strategies are likely to produce a positive effect, as a large amount of time devoted to volunteering activities is a predictor of burnout [59]. A recent study has suggested  that people’s sense of community commitment is often a reason for sustained engagement and that cohesive community relationships are particularly relevant for continuous volunteering over time [47]. Yet, and despite the extensive literature on the factors influencing participation in volunteering [60], the predictors of volunteering during the pandemic may be slightly different from others forms of volunteering [38]. Hence, further research is needed to understand how COVID-19 community groups can be sustained over time, by examining the role of these structural, psychological and contextual variables.

Many factors were identified as fostering effective volunteering endeavours, including local knowledge, existing relationships and trust built up over the years by pre-existing organisations were crucial to enable effective large-scale responses [32, 39]. Despite its successes and achievements, volunteer groups faced several challenges, with this review suggesting some important lessons on how to foster engagement with local communities during the COVID-19 period. In this regard, studies reviewed highlight the need for local councils to improve the way support is provided to volunteering groups and other community-based organisations [61]. Such support is key for community engagement in pandemic conditions, and as a way to reduce contagion [10–12, 62].

The COVID context has changed a lot since October 2020 when this rapid review was carried out, and it continues to evolve. In the UK, after a decline in cases over the summer 2020, there were two further waves in the autumn 2020 and January 2021, with a seven-day daily average of over 50,000 cases for a period of time. This rise in cases translated into many people requiring support to stay at home. When cases came down in the late winter and early spring (coinciding with both ‘lockdown’ and the successful roll out of the vaccine) the UK government introduced a mass testing programme [63], which again led to numbers of people having to self-isolate, some without symptoms. The evidence that rates of self-isolation were low in the UK led to a number of government interventions to provide greater financial support, including a £500 payment for people below a certain level of income who were self-isolating (September 2020), a discretionary fund of £20 million per month and a £3.2 million per month medicine delivery service, both announced in March 2021 [16]. The most recent large survey [22] identified some significant improvements in levels of self-isolation but concluded that levels were still too low (duration adjusted adherence to full self-isolation was 42.5% of people) and that ‘practical support and financial reimbursement are likely to improve adherence’. This suggests very strongly that, in the absence of ‘wrap-around’ services as seen in some countries [13], the activity of community volunteers will remain vital until the pandemic is over. It is therefore still important to understand the factors – both practical and psychological – that sustain such volunteering.

In particular, further research should focus on how organisations, official and non-official bodies, can improve their collaboration and cooperation with volunteer-based groups, in order to foster community engagement and participation. Community champions schemes may have the potential to foster community engagement [19], but research is needed to understand the role of community champions and the role of official organisations in facilitating and fostering community-based volunteering during COVID-19. Additionally, providing more evidence on how communities can be effectively engaged in decision-making during and beyond this pandemic is also crucial.

## Conclusions

The purpose of this rapid review was to expand our understanding of and map current literature exploring volunteering within the context of COVID-19. This review advances understanding of the nature and dynamics of COVID-19 volunteering as well as of how community engagement was fostered during the COVID-19 pandemic in the UK. Overall, our review suggests that there were diverse models of organisation and coordination in COVID-19 volunteering and that community support groups adjusted their activities and scope of action to perceived needs and challenges. Social networks and connections, local knowledge, and social trust were key dimensions associated with community organising and volunteering. Additionally, volunteers were mostly women, middle-class, highly educated, and working-age people. Notably, our review also suggests that there has been limited community engagement and collaboration with volunteering groups and other community-based organisations. Considering the importance of public engagement and community support in pandemic conditions [10], and in public health more generally [64], this review is relevant for interventions far beyond the current COVID-19 pandemic.

## Data Availability

Not applicable.

## Abbreviations

DBS: Disclosure and Barring Service
HEART: Hastings Emergency Action Response Team
NHS: National Health Service
PRISMA: Preferred Reporting Items for Systematic Reviews and Meta-Analyses
WHO: World Health Organization
NIPs: Non-Pharmaceutical Interventions
MMAT: Mixed Methods Appraisal Tool
NCVO: National Council for Voluntary Organisations

## Footnotes

1 Someone who tested positive for COVID-19 must self-isolate for 10 days. If in contact with someone that has symptoms or has tested positive, the recommendation is to self-isolate for 14 days [10].
